# Sand fly endosymbionts in Kenya: *Rickettsia* and *Wolbachia* associations with *Leishmania* and detection of *Rickettsia africae*

**DOI:** 10.1186/s13071-026-07283-7

**Published:** 2026-02-11

**Authors:** Steve Kiplagat, Damaris Matoke-Muhia, Barrack O. Owino, David P. Tchouassi, Daniel K. Masiga, Gregory D. D. Hurst, Jandouwe Villinger

**Affiliations:** 1 International Centre of Insect Physiology and Ecology (icipe), P.O. Box 30772-00100, Nairobi, Kenya; 2 Centre for Biotechnology Research and Development, Kenya Medical Research Institute, Nairobi, Kenya; 3 Department of Biological and Medical Sciences, Oxford Brookes University, Gipsy Lane, Oxford, OX3 0BP UK; 4 Institute of Infection, Veterinary and Ecological Sciences, University of Liverpool, Liverpool, UK

**Keywords:** Endosymbionts, Gut bacteria, Sand flies, Rickettsia, Leishmania

## Abstract

**Background:**

Sand flies are small hematophagous insects known as leishmaniasis vectors. Similar to most arthropods, they harbor nonobligate endosymbionts that may influence host adaptation and pathogen transmission, but these symbiont communities remain poorly characterized in *Leishmania*-endemic African sand flies.

**Methods:**

We screened 1700 wild-caught phlebotomine sand flies (1266 females, 434 males) from Kenya’s Baringo, Nakuru, and Kajiado counties, and 253 colony *Phlebotomus duboscqi*, for *Rickettsia*, *Wolbachia*, *Spiroplasma*, *Cardinium*, *Arsenophonus*, Microsporidia, and *Leishmania* by high-resolution melting analysis and sequencing of PCR products.

**Results:**

In wild sand flies (*Phlebotomus* and *Sergentomyia* spp.), *Wolbachia* spp. were most common (8.5%, 145/1700), with particularly high prevalences in *Ph. mireillae* (92.3%, 12/13), *Ph. guggisbergi* (73.2%, 82/112), and *Ph. saevus* (48.6%, 18/37), followed by *Spiroplasma* (1.4%, 23/1700), *Rickettsia* (0.7%, 12/1700), *Cardinium* (0.4%, 6/1700), *Tubulinosema* sp. (0.1%, 1/1700), and various gut bacteria (1.8%, 30/1700). In addition, we detected *Rickettsia africae*, a tick-borne pathogen causing African tick-bite fever (ATBF), in *Ph. martini* (4.7%, 5/106), *Ph. guggisbergi* (1.8%, 2/112), *S. schwetzi* (0.4%, 1/263), *S. clydei* (0.5%, 2/440), and *Sergentomyia* sp. (0.3%, 1/371). Notably, *R. africae* DNA was found in one male *Ph. martini* and *Rickettsia* sp. DNA in one male *S. clydei* and one male *S. schwetzi*, consistent with infection rather than blood-meal contamination. Furthermore, *Rickettsia* endosymbionts were positively associated with *Leishmania* DNA (OR = 20.31; 95% CI [4.93, 77.03]; *P* < 0.0001), including within *Phlebotomus* (OR = 13.54; 95% CI [2.33, 78.88]; *P* = 0.0017). *Wolbachia* also correlated with *Leishmania* overall (OR = 2.46; 95% CI [1.17, 4.79]; *P* = 0.011), though not within individual fly genera. Colony *Ph. duboscqi* harbored only *Serratia* and other gut bacteria.

**Conclusions:**

Sand flies in Kenya harbored six endosymbionts, including the first detection of pathogenic *R. africae* in sand flies, and gut bacteria that may influence vector competence. The frequent co-occurrence of *Rickettsia* and *Wolbachia* endosymbionts with *Leishmania* indicates nonrandom associations between symbionts and parasite infection, without implying causality. These findings reveal previously undescribed sand-fly–microbe interactions, and highlight the need for experimental studies to test whether sand flies contribute to the ecology and potential transmission of ATBF.

**Graphical Abstract:**

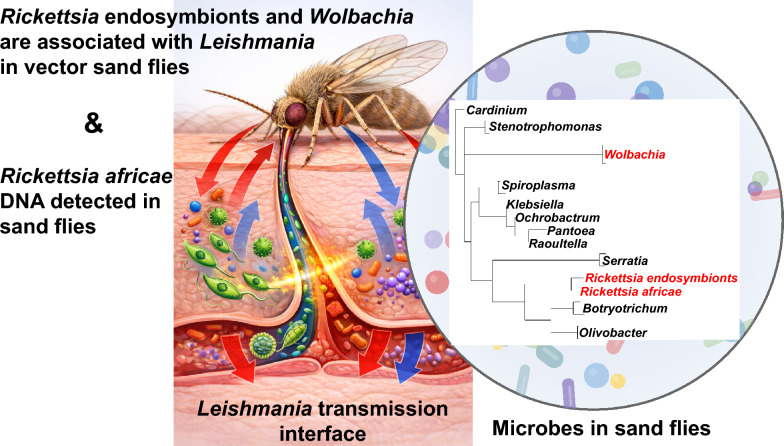

**Supplementary Information:**

The online version contains supplementary material available at 10.1186/s13071-026-07283-7.

## Background

Sand flies (Diptera: Psychodidae) are insects of medical importance because of their role in the transmission of various agents of human and veterinary disease [1], including phleboviruses, *Bartonella bacilliformis*, and most importantly, *Leishmania* (Kinetoplastida: Trypanosomatidae) parasites, which infect and cause human disabling disease leishmaniasis [1, 2]. Emergence of leishmaniasis outside known endemic areas [3], including complex transmission dynamics recently documented in Kenya [4–7], along with lack of vaccines, emergence of drug resistance, and paucity of new drugs against leishmaniasis [8] has created an imperative for novel disease mitigation approaches, including vector control strategies [9, 10].

Since the discovery of insect gut bacteria with promising impacts in arthropods against vector-borne disease [11–13], microbial endosymbionts of sand flies have gained attention owing to their potential impact on sand fly fitness and growth of *Leishmania* parasites in host sand flies [14]. They commonly include a range of bacteria and fungi such as *Rickettsia* (Rickettsiales: Rickettsiaceae), *Wolbachia* (Rickettsiales: Rickettsiaceae), *Cardinium* (Bacteroidales: Bacteroidaceae), *Spiroplasma* (Entomoplasmatales: Spiroplasmatacae), *Arsenophonus* (Enterobacterales; Morganellaceae), and microsporidia [15]. These microbes are of interest as they can play significant biological roles in controlling disease transmission by either modulating host insect behavior, physiology, or conferring resistance to pathogens and parasites development in the host [16]. Here, we distinguish between vertically transmitted (“heritable”) endosymbionts, such as *Rickettsia*, *Wolbachia*, *Spiroplasma*, *Cardinium*, and environmentally acquired or transient gut-associated bacteria, which may also modulate sand fly biology but are less likely to be inherited.

In arthropods, endosymbionts of the alphaproteobacterial order Rickettsiales include five major genera of importance as agents of disease or as symbionts of arthropods that spread disease: *Anaplasma*, *Ehrlichia*, *Orientia*, *Rickettsia*, and *Wolbachia*. Members of these taxa infect diverse eukaryotic hosts, including humans, livestock, insects, and protists [17]. They are gram-negative bacteria, some of which have a spectrum of endosymbiotic relationships from parasitism to obligatory mutualism in a wide range of arthropods [18]. *Wolbachia*, for example, has evolved strategies to manipulate host reproduction, including parthenogenesis, feminization, male killing, and cytoplasmic incompatibility to facilitate their own propagation, proliferation, transmission, and others provide an additional fitness benefit for the host to protect against pathogens [18, 19]. Successes in the experimental transinfection of selected strains of *Wolbachia* into mosquito species such as*Aedes aegypti* have been deployed to prevent transmission of dengue viruses, preventing dengue and Zika virus transmission by either inhibiting binding of the viruses to mosquito cells, directly blocking the virus, or by decreasing the lifespan of the vector [20, 21].

Similar to *Wolbachia*, *Rickettsia* have been found to influence host fitness by manipulating reproduction to enhance their transmission and cause thelytokous parthenogenesis (where mothers produce daughters from unfertilized eggs) in parasitoid wasp and *Pnigalio soemius* (Hymenoptera: *Eulophidae*) [22] and male-killing in the two spot ladybird *Adalia bipunctata* [23]. Both *Rickettsia* and *Wolbachia* are primarily transmitted vertically to host progeny, across the generations [22, 24], and along with *Wolbachia*, these two symbionts have the potential to enable biological control strategies against vectors of diseases and their pathogens [24].

Recent research has highlighted the role of bacterial symbionts in vectors of medical importance. A notable discovery is the microsporidian species, microsporidia MB, which impairs the development and transmission of *Plasmodium* in *Anopheles arabiensis* mosquitoes [25]. Remarkably, this impairment occurs without significantly affecting the host’s fertility, fecundity, development, or longevity, making this taxon an appealing candidate for pathogen blocking [26]. Consequently, there is need for more research to identify further endosymbionts relevant for innovative biocontrol strategies to curb pathogen transmission [27], especially in sand flies where most of the impactful bacterial endosymbionts remain largely unexplored [28].

To understand the effects and role of endosymbionts on the growth and development of *Leishmania* and the ecological evolution of their hosts, we screened sand flies collected in three study areas of Kenya’s Rift Valley region for diverse endosymbionts, determined their occurrence, prevalence, and diversities, and investigated potential associations of specific endosymbionts with naturally occurring *Leishmania* parasites. To our knowledge, this is the first study to jointly characterize multiple heritable endosymbionts, gut bacteria, *Leishmania* parasites, and the tick-borne *R. africae*, a zoonotic tick-borne pathogen responsible for African tick bite fever (ATBF), in sand flies from Kenya, and to quantify statistical associations between these microbes.

## Methods

### Study areas and sand fly collections

**Fig. 1:**
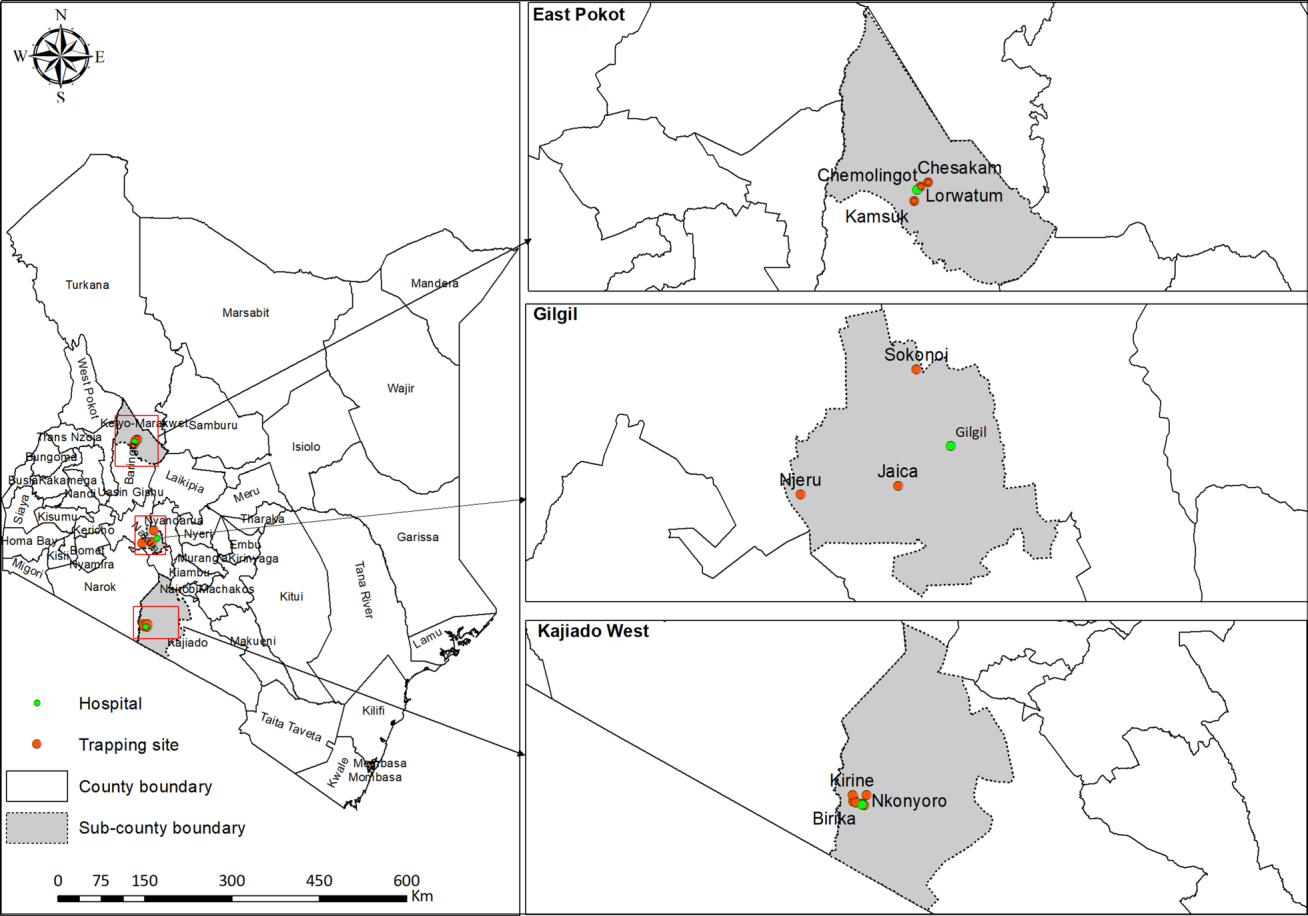
Sampling sites of Kenyan sand flies. East Pokot (Top) and Gilgil (Middle) in Baringo and Nakuru counties lie in the upper Rift Valley, whereas Kajiado West Sub-County (bottom) in Kajiado County lies in the lower Rift Valley. Temporary field laboratories were established at Chemolingot Dispensary, Gilgil Sub-County Hospital, and icipe’s field station at Nguruman (Green)

We collected sand flies from three study areas in the upper (East Pokot and Gilgil sub-counties) and lower (Kajiado West sub-county) parts of Kenya’s Rift Valley. We selected the study areas and sites (Additional file Supplementary1: Table S1) on the basis of active endemic areas of both cutaneous (CL) and visceral (VL) forms of leishmaniasis in Kenya. The trapping sites included Chesakam, Lorwatum, and Kamsuk in Chemolingot in East Pokot sub-county (Baringo County, 0.01419^o^E, 36.01557^o^N), Thugunui, Njeru, and Jaica in Gilgil sub-county (Nakuru County −0.582909^o^E, 36.083596^o^N), and Nguruman; Birika, Nkonyoro, Olosinyai, Olomanyatta, Empaleki, Enchanipus, Kirine, and Shompole in Kajiado West sub-county (Kajiado County −1.875549^o^S, 36.157326^o^N) (Fig. 1 and Additional file Supplementary1: Table S1). Generally, with exception of Gilgil sub-county where rock crevices were covered by green shrubs, acacia trees were predominant vegetation in all the areas along with shrubs and dry grass. These habitat types are known to influence sand fly population structure in Kenyan leishmaniasis foci [7]. We trapped sand flies predominantly from termite mounds in East Pokot and Kajiado West, while in Gilgil we set sand fly traps in rock crevices, caves, and animal burrows inhabited by rock hyraxes, bats, and rodents, respectively.

Sand fly collection in East Pokot also involved trapping sand flies in caves and rock crevices in hillocks of volcanic origin. We also sampled sand flies in peridomestic environments, such as nearby animal sheds and the periphery of homesteads, using standard Center for Disease Control (CDC) miniature light traps between 6PM and 7AM the following day. The samples were transported to a temporary field laboratory where we processed sand flies for dissections, morphological identification and preservation in Liquid Nitrogen awaiting transportation to Emerging Infectious Diseases (EID) laboratory at *icipe*’s Duduville campus in Nairobi, Kenya, where they were stored at −80 °C freezers awaiting DNA extraction for subsequent molecular screening and identification.

### Sand fly dissections and morphological identification

After sand fly processing that involved sorting and washing in 2% teepol detergent with subsequent washing in distilled water twice, we dissected out the head and the genitalia in each sand fly for morphological identification while the thorax and the remaining part of the abdomen was preserved in liquid nitrogen for later molecular analysis. We mounted the sand fly parts dissected out onto a labeled microscope slide using gum chloral-hydrate (locally prepared Hoyer’s mounting medium) on dissecting microscope and kept in a room temperature surface to dry. Morphologically, we identified each sand fly using combination of phlebotomine dichotomous keys [29, 30] using Olympus CX31 compound microscope.

### Rearing of *Ph. duboscqi* sand flies

All the *Phlebotomus duboscqi* specimens used in this study were bred at *icipe*’s sand fly rearing unit. Sand flies were maintained on apple fruits until they were 2–3 weeks old under laboratory condition of temperature between 26–28 °C and 75–80% humidity.

### Extraction of genomic DNA from sand flies

In the laboratory, we homogenized each preserved sand fly body (thorax with part of abdomen) in 180 µl of buffer ATL (Qiagen GmbH, Hilden, Germany). We extracted the genomic DNA from each homogenate using DNeasy Blood and Tissue Kit (Qiagen GmbH, Hilden, Germany) following the manufacturers’ instructions. We eluted obtained DNA in 30 µl of buffer ET and stored at −20 °C for later use.

### Molecular identification of sand flies

We verified morphological sand fly species identifications by amplifying sand fly cytochrome oxidase 1 gene (CO1) (Table 1) using COI primers and *cytochrome b* gene (*cyt b*) using *cyt b* primers (Table 1), which yield DNA amplicons sizes of about 700 bp and 386 bp, respectively. The two sand fly biomarkers (COI and *cyt b* regions) were amplified in 20-µl PCR reactions that included 4 µl of X5 Blend (Biodyne, Solis) ready-to-load master mix, 1 µl of each specific primers, and 12 µl of nuclease free water (Sigma, St. Louis, USA). We used 2 µl (2.5–7.0 ng/ µm) of each template DNA.

**Table 1:** List and description of primers used in this study

Target organism	Primer name	Primer sequence	Annealing (°C) & (No. of cycles)	Cycling conditions	Amplicon size (bp)	Reference
*Wol bachia*	wsp81F	TGGTCCAATAAGTGATGAAGAAAC	55 (35)	Denaturation: 95 °C for 30 s; Annealing: 45 s; Extension: 72 °C for 60 s	550	[32, 33]
	wsp691R	AAAAATTAAACGCTACTCCA				
	wspecF	CATACCTATTCGAAGGGATAG	62 (35)	Denaturation: 95 °C for 30 s; Annealing: 30 s; Extension: 72 °C for 40 s	440	[34]
	wspecR	AGCTTCGAGTGAAACCAATTC				
*Rickettsia*	Rick 16S F	GAACGCTATCGGTATGCTTAACACA	51 (35)	Denaturation: 95 °C for 30 s; Annealing: 45 s; Extension: 72 °C for 30 s	365/500	[35]
	Rick 16S R	CATCACTCACTCGGTATTGCTGGA				
	rOmpB 120–2788	AAACAATAATCAAGGTCATGT	53 (35)	Denaturation: 95 °C for 30 s; Annealing: 30 s; Extension: 72 °C for 40 s	856	[31]
	rOmpB 120–3599	TACTTCCGGTTACAGCAAAGT				
Microsporidia	Msp SSU RNA F	CACCAGGTTGATTCTGCC	62 (35)	Denaturation: 95 °C for 45 s; Annealing: 60 s; Extension: 72 °C for 90 s	600	[25]
	Msp SSU RNA R	TTATGATCCTGCTAATGGTTC				
	Msp SSU RNA F 1492R	CACCAGGTTGATTCTGCC GGTTACCTTGTTACGACTT	60 (35)	Denaturation: 95 °C for 30 s; Annealing: 60 s; Extension: 72 °C for 60 s	1200	[36]
*Cardinium*	Car-sp-F	CGGCTTATTAAGTCAGTTGTGAAATCCTAG	57 (35)	Denaturation: 95 °C for 30 s, Annealing: 40 s Extension: 72 °C for 60 s	544	[37]
	Car-sp-R	TCCTTCCTCCCGCTTACACG				
*Spiroplasma*	SpoulF	GCTTAACTCCAGTTCGCC	55 (35)	Denaturation: 95 °C for 30 s; Annealing: 60 s; Extension: 72 °C for 60 s	440	[38]
	SpoulR	CCTGTCTCAATGTTAACCTC				
*Arsenophonus*	ArsF	GGGTTGTAAAGTACTTTCAGTCGT	54 (35)	Denaturation: 95 °C for 30 s; Annealing: 30 s; Extension: 72 °C for 45 s	1200	[39]
	ArsR2	GTAGCCCTRCTCGTAAGGGCC				
	Ars-23S-1	CGTTTGATGAATTCATAGTCAAA	60 (35)	Denaturation: 95 °C for 30 s; Annealing: 45 s; Extension: 72 °C for 60 s	600	[40]
	Ars-23S-2	GGTCCTCCAGTTAGTGTTACCCAAC				
Insect COI	LepF1	ATTCAACCAATCATAAAGATATTGG	45 and 51 (5 and 35)	Denaturation: 95 °C for 40 s; Annealing: 45 °C for 40 s and 51 °C for 40 s; Extension: 72 °C for 60 s	704	[41]
	LepR1	TAAACTTCTGGATGTCCAAAAAATCA				
Insect *cyt b*	Cytb-J-1–933	TCTTTTTGAGGAGCWACWGTWATTAC	45 (35)	Denaturation: 95 °C for 60 s; Annealing: 60 s; Extension: 72 °C for 120 s	386	[42]
	Cytb-N-11367	AATTGAACGTAAAATWGTRTAAGCAA				
*Leishmania*	L5.8S	TGATACCACTTATCGCACTT	58 (35)	Denaturation: 95 °C for 20 s; Annealing: 30 s; Extension: 72 °C for 30 s	365	[5, 43]
	LITSR	CTGGATCATTTTCCGATG				

PCR conditions for *cyt b* amplification included an initial denaturation at 95 °C for 15 min, followed by 35 cycles of denaturation at 95 °C for 1 min, followed by annealing at 45 °C for *cyt b* for 1 min, extension at 72 °C for 2 min, and a final extension at 72 °C for 7 min. The COI PCR conditions included initial denaturation at 95 °C for 5 min, followed by five cycles with denaturation at 95 °C for 40 s, annealing at 45 °C for 40 s and extension at 72 °C for 1 min, and 35 cycles with denaturation at 95 °C for 40 s, annealing at 51 °C for 40 s, and extension at 72 °C for 1 min, and a final extension at 72 °C for 7 min. We used ProFlex PCR System (ThermoFischer Scientific Inc., USA), or the Kyratec Supercycler (POCD Scientific, Australia) in our subsequent PCR analysis.

### PCR analysis and identification of specific endosymbionts in sand flies

To identify sand flies with specific endosymbionts, *Wolbachia*, *Cardinium*, *Spiroplasma*, *Arsenophonus*, and microsporidia, we screened whole sand fly genomic DNA using specific primers under their specific PCR conditions (Table 1) using Proflex or Kyratech thermocyclers. In total, we screened genomic DNA from all 1700 wild-caught sand flies (1266 females and 434 males) and 253 colony *Ph. duboscqi* for these endosymbionts and *Rickettsia*.

To improve the sensitivity for *Rickettsia* detection, we first used high-resolution melting (HRM) analysis of rickettsial 16S rRNA gene PCR products using Magnetic Induction Cycler (MIC) (Bio Molecular Systems, Queensland, Australia) machines [31]. *Rickettsia* with unique rickettsial 16S rRNA HRM profiles were further characterized by sequencing of the citrate rickettsial outer membrane protein B (*ompB*) gene PCR products [31].

We used 2 µl (2.5–7.0 ng/ µm) of each DNA as template in a master mix containing 2 µl of 5X Hot FIREPol Blend or HRM (Biodyne, Solis) ready-to-load master mixes, 1 µl of each specific primers, and nuclease free water (Sigma, St. Louis, USA). We used nuclease free water (Sigma, St. Louis, USA) also as negative control in each of our subsequent PCRs. PCRs were performed under each specific PCR condition, depending on target endosymbiont primers, that included initial denaturation at 95 °C for 2–15 min, followed by 35–40 cycles that included denaturation at 95 °C for 20–60 s, annealing temperatures (45–62 °C depending on each primer; Table 1), extension at 72 °C for 30–120 s and final extension at 72 °C for 5–7 min.

### Detection and identification of *Leishmania* parasites in sand flies

We screened for *Leishmania* parasites in sand flies by amplifying internal transcribed spacer 1 gene (*ITS1*) using L5.8S and LITSR primers (Table 1). We screened 1266 wild-caught females (all morphologically identified females from the 1700 field-collected sand flies) for *Leishmania* DNA; males were not tested for *Leishmania*. We used 2 µl sample DNA of 2.5–7.0 ng/µm as template in a master mix with 2 µl of 2X Dream Taq Green PCR Master Mix, 0.5 µl of each primer and nuclease-free water (Sigma, St. Louis, USA). We used *L. donovani* DNA (NLB65), *L. major* DNA (LD63), and *L. tropica* DNA as positive controls and nuclease free water (Sigma, St. Louis, USA) as negative control in our subsequent PCRs. We performed PCRs under cycling conditions that involved initial denaturation at 98 °C for 2 min followed by 35 cycles of denaturation, annealing, and extension as indicated in Table 1, and a final extension at 72 °C for 7 min. We used Proflex or Kyratech thermocyclers in our PCR analysis.

We resolved all obtained amplicons in 1.5% agarose gels stained with × 1 ethidium bromide and visualized in Kodak Gel Logic 200 Imaging System (SPW Industrial, Laguna Hills, CA, USA). Amplicons of expected DNA band sizes were purified using the QIAquick PCR purification kit (QIAGEN, CA. USA) according to manufacturers’ protocol and submitted for Sanger sequencing using forward and reverse primers.

### Sequencing analyses

We analyzed the obtained nucleotide sequence chromatograms using the MAFFT plugin in Geneious Prime software v. 2023.1.2. Each consensus sequence was queried for related reference sequences in the GenBank database using the Basic Local Alignment Search Tool (www.ncbi.nlm.nih.gov/BLAST/). A similarity of over 99% in each queried sequence against the subject sequences at the GenBank database was considered to be the most likely organism. Maximum-likelihood phylogenies of the studied sand fly species based on the organism target gene sequences, aligned with reference sequences retrieved from GenBank, were constructed using PhyML v. 3.0, with automatic model selection on the basis of the Akaike information criterion [44]. We estimated Tree topologies over 1000 bootstrap replicates with the nearest neighbor interchange improvements [45]. We visualized our Phylogenetic trees using FigTree v. 1.4.4 [46].

### Data analysis

To understand the modulatory effects of endosymbionts to *Leishmania* parasite development in sand flies, we used a Fisher’s exact test to test for associations between *Leishmania* and endosymbionts in both *Phlebotomus* and *Sergentomyia* sand flies and also in each sand fly species considering *P*-values < 0.05 as statistically significant. All our data were entered in excel sheet v2006 and analyzed in R software v4.2.2. Prevalence estimates were calculated as the proportion of positive specimens out of the total screened, and 95% confidence intervals (95% CIs) were obtained using the Clopper–Pearson exact binomial method.

An overview of the laboratory screening workflow (from collection and morphological identification through DNA extraction, PCR-HRM screening and sequencing) is provided in Additional file 2: Supplementary Fig. S1.

## Results

### Morphological identification of sand flies

We collected 1700 sand flies (1266 females and 434 males) comprising 290 (17.1%) *Phlebotomus* spp. and 1410 (82.9%) *Sergentomyia* spp. from the three study areas (Table 2). We morphologically identified seven *Phlebotomus* and nine *Sergentomyia* sand fly species. *Phlebotomus* sand flies included *Ph. aculeatus* (0.1%, *n* = 1), *Ph. duboscqi* (0.2%, *n* = 3), *Ph. guggisbergi* (6.6%, *n* = 112), *Ph. martini* (6.2%, *n* = 106), *Ph. mireillae* (0.8%, *n* = 13), *Ph. orientalis* (1.1%, *n* = 18), and *Ph. saevus* (2.2%, *n* = 37). *Phlebotomus martini* was collected in Kajiado West and East Pokot sub-counties, while both *Ph. saevus* and *Ph. guggisbergi* were collected in areas of Kajiado West and Gilgil sub-counties. *Phlebotomus orientalis* was found only in Kajiado West. *Phlebotomus aculeatus* and *Ph. mireillae* were collected only in Gilgil, while *Phlebotomus duboscqi* sand flies were only found in areas of East Pokot in Baringo County. Sand flies of the genus *Sergentomyia* were commonly found in all the areas and included *Sergentomyia* sp*.* (21.8%, *n* = 371), *Sergentomyia schwetzi* (15.5%, *n* = 263), *Sergentomyia africana* (2.5%, *n* = 43), *Sergentomyia antennata* (4.4%, *n* = 74), *Sergentomyia adleri* (7.7%, *n* = 130), *Sergentomyia bedfordi* (4.7%, *n* = 79), *Sergentomyia dreyfussi* (0.1%, *n* = 1), and *Sergentomyia ingrami* (0.5%, *n* = 9). In addition, 253 (198 females and 55 males) colony *Ph. duboscqi* sand flies were also screened for heritable endosymbionts.

**Table 2:** Sand fly composition and diversity in Kenya’s Baringo, Nakuru, and Kajiado Counties

Sand fly species	East Pokot, Baringo County			Gilgil, Nakuru County			Kajiado West, Kajiado County								Total
	Chesakam	Kamsuk	Lorwatum	Njeru	Jaica	Thugunui	Birika	Empaleki	Enchanipus	Kirine	Olomanyatta	Oloisinyai	Nkonyoro	Shompole	
*Ph. aculeatus*	0	0	0	1	0	0	0	0	0	0	0	0	0	0	1
*Ph. duboscqi*	2	0	1	0	0	0	0	0	0	0	0	0	0	0	3
*Ph. guggisbergi*	0	0	0	12	30	69	0	0	0	0	0	0	1	0	112
*Ph. martini*	2	2	7	0	0	0	0	9	7	25	22	24	3	5	106
*Ph. mireillae*	0	0	0	13	0	0	0	0	0	0	0	0	0	0	13
*Ph. orientalis*	0	0	0	0	0	0	0	0	2	2	5	9	0	0	18
*Ph. saevus*	0	0	0	0	0	0	0	0	0	0	0	0	37	0	37
*S. adleri*	0	0	0	0	0	0	0	7	16	1	20	10	2	74	130
*S. africana*	8	12	3	0	0	0	1	0	4	10	1	1	0	3	43
*S. antennata*	20	0	0	0	0	0	5	0	2	4	7	4	25	7	74
*S. bedfordi*	4	4	4	0	0	0	1	0	5	14	3	2	7	35	79
*S. clydei*	31	0	20	0	0	0	1	39	66	38	183	30	23	9	440
*S. ingrami*	0	0	0	0	0	0	0	0	0	0	0	0	0	9	9
*S. schwetzi*	118	80	56	0	0	0	0	0	0	9	0	0	0	0	263
*Sergentomyia* sp.	10	1	11	0	0	0	0	6	64	24	38	184	26	7	371
*S. dreyfussi*	1	0	0	0	0	0	0	0	0	0	0	0	0	0	1
Total:	196	99	102	26	30	69	8	61	166	127	279	264	124	149	1700
%:	11.5	5.8	6.0	1.5	1.8	4.1	0.5	3.6	9.8	7.5	16.4	15.5	7.3	8.8	100

### Molecular identification and phylogenetic analysis of sand flies

**Fig. 2:**
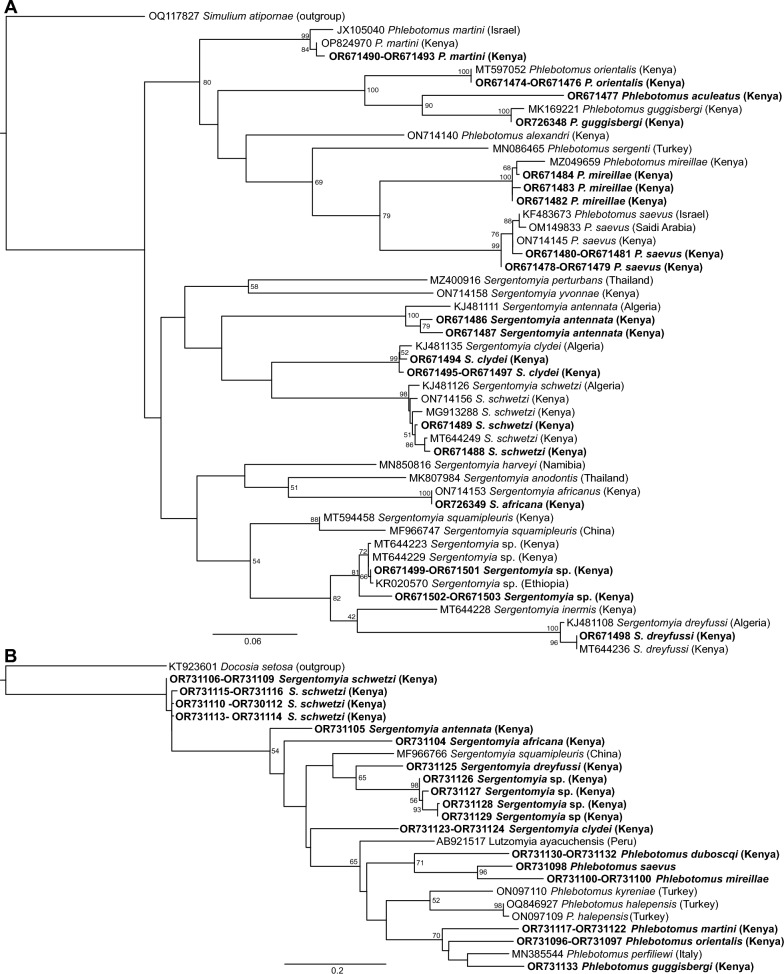
Maximum-likelihood trees of sand fly **A** COI and **B** *cyt b* gene sequences. Sequences from this study are in bold. Bootstrap support values (percentages) are shown at major nodes (1000 replicates). Scale bar represents substitutions per site

Phylogenetically, the two cryptic sand flies of the *Paraphlebotomus* subgenus identified, *Ph. saevus* (GenBank accessions: COI OR671478 to OR671481) and *Ph. mireillae* (GenBank accessions: COI OR671482-OR671484; *cyt b* OR731100 to OR731103), shared 99% similarity with sequences of *Ph. saevus* from Israel (GenBank: KF483673) and *Ph. mireillae* previously sequenced from Gilgil, Kenya (GenBank: MZ049659), respectively (Fig. 2). As expected, sequences of *Ph. martini* (GenBank accessions: OR671490 to OR671493; *cyt b* OR731117 to OR731122) from this study shared 98.5% similarity with those of *Ph. martini* (GenBank: JX105040) and *Ph. celiae* from Israel (GenBank: JX105041), while *Ph. guggisbergi* sequence (GenBank accessions: COI OR726348; *cyt b* OR731133) shared 99% similarity with another *Ph. guggisbergi* sequence from Kenya (GenBank: MK169221). Sequences of *Ph. orientalis* (GenBank accessions: COI OR671474 to OR671476; *cyt b* OR731096 and OR731097) shared 99% identity *Ph. orientalis* from Ethiopia (KC204967) and Kenya (MT597052).

The *Ph. aculeatus* (GenBank accession: COI OR671477) was more phylogenetically related to sequences of *Ph. guggisbergi* than to those of *Ph. orientalis* (Fig. 2A). Sequences of *Sergentomyia* sp. (GenBank accessions: COI OR671499-OR671503; *cyt b* OR731126 to OR731129) shared 99.5–100% identity with *Sergentomyia* sp. sequences from Kenya (MT644223 and MT644229) and Ethiopia (KR020570), while *S. dreyfussi* (GenBank accessions: COI OR671498; *cyt b* OR731125) shared 100% identity with a previously sequenced *S. dreyfussi* from Kenya (MT644236).

### Molecular analysis of bacterial symbionts and pathogens

**Fig. 3:**
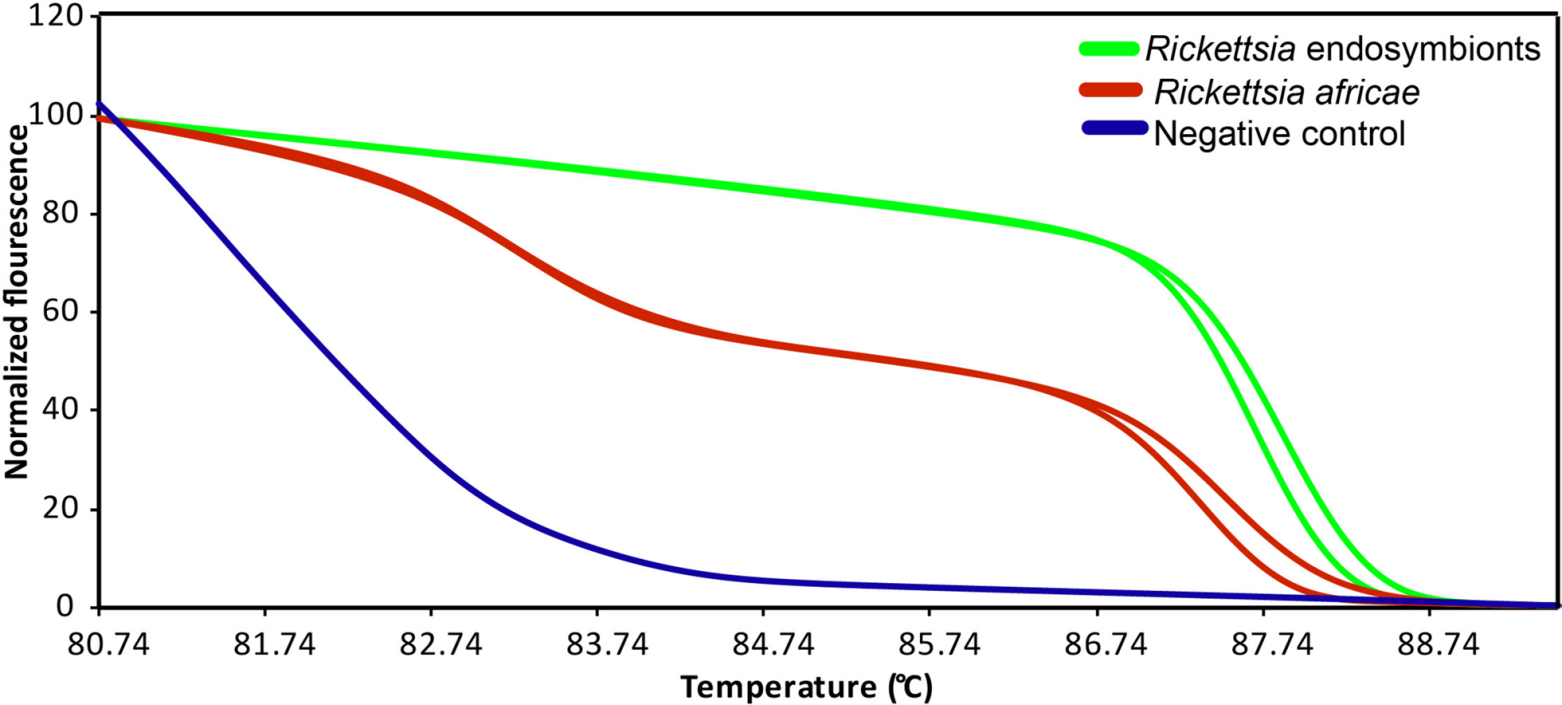
Normalized high-resolution melting (HRM) profiles of *R. africae* and *Rickettsia* endosymbiont 16S rRNA gene amplicons

**Fig. 4:**
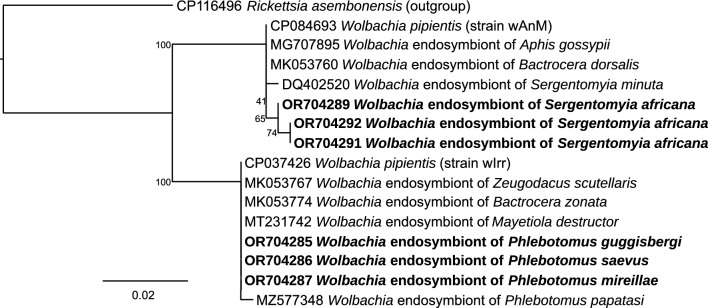
Maximum-likelihood trees of **A** *Rickettsia* 16S rRNA, **B** *Rickettsia ompB*, and **C** *Wolbachia* 16S rRNA sequences. Sequences from this study are shown in bold. Bootstrap support values (percentages) from 1000 replicates appear at major nodes. Scale bar indicates substitutions per site

Using HRM analysis and representative 16S rRNA amplicon sequencing, we identified *Wolbachia* endosymbionts in *Phlebotomus* (GenBank accessions OR704218-OR704288, OR704290) and *Sergentomyia* (GenBank accessions OR704289, OR704291, OR704292) sand flies*, Rickettsia* spp. endosymbionts (GenBank accessions OR704173-OR704178), *Spiroplasma* sp. (GenBank accessions OR704211-OR704217), *Serratia* sp*.* (GenBank accessions OR704204-OR704210), *Cardinium* sp. (GenBank accession OR704293), and *Tubulinosema* sp. (GenBank accession OR717175). We also identified *Rickettsia africae* (GenBank accessions: 16S rRNA OR704165-OR704172). The distinct HRM profiles of the *R. africae* and *Rickettsia* endosymbionts are shown in Fig. 3. The *R. africae* 16S rRNA sequences shared 100% similarity with *R. africae* sequences accessed from GenBank (Fig. 4A). Representative *R. africae ompB* gene sequences from this study (GenBank accessions OR731090, OR731091) also shared 100% similarity with *R. africae* sequences obtained from GenBank (Fig. 4B). The *Rickettsia* spp. endosymbiont sequences clustered phylogenetically with *Rickettsia* endosymbionts of other insect species (Fig. 4A).

The *Wolbachia* 16S rRNA sequences amplified from *Phlebotomus* sand flies shared 100% sequence identity with *Wolbachia* endosymbionts of *Phlebotomus papatasi* (GenBank accession MZ577348) and other insects, whereas those sequenced from *Sergentomyia* sand flies shared 100% sequence identity with a *Wolbachia* endosymbiont of *Sergentomyia minuta* (GenBank accession DQ402520) and other insects (Fig. 4C). In the 16S rRNA phylogeny, *Wolbachia* sequences from *Phlebotomus* clustered with the wPap strain from *Ph. papatasi* (supergroup A), whereas those from *Sergentomyia* grouped with *Wolbachia* from *Se. minuta* (supergroup B), indicating that Kenyan sand flies host at least these two major *Wolbachia* supergroups, although 16S alone cannot fully resolve strain diversity.

The *Spiroplasma* sp. sequences shared 99% identity with *Spiroplasma citri* (KR818831) from Malaysia. We obtained only one sequence of *Cardinium* sp. endosymbiont that shared > 97% identity with a *Cardinium* endosymbiont of *Microzetorchestes emeryi* (LC090049) from Spain. We also found a microsporidian, *Tubulinosema* sp. (GenBank accession OR717175), for the first time in sand flies, which shared 100% sequence similarity with sequences of *Tubulinosema acridophagus* from a human patient with microsporidiosis from Belgium (JQ247017) [47] and *Tubulinosema acridophagus* from a mosquito colony collapse in Argentina (AF024658).

Along with endosymbionts, we amplified gut bacteria DNA of ten genera: *Acetobacter* (*n* = 3), *Botryotrichum* (*n* = 1), *Enterobacter* (*n* = 1), *Pantoea* (*n* = 2), *Halomonas* (*n* = 1), *Klebsiella* (*n* = 3), *Olivibacter* (*n* = 1), *Stenotrophomonas* (*n* = 2), *Rhizobiales* (*n* = 1), and *Asaia* sp. (*n* = 1) bacteria (Table 3), using the *Arsenophonus* primers. As summarized in Additional file 1: Supplementary Table S2 (including all microbes found in this study), sequences of gut bacteria shared similarities to their corresponding GenBank sequences. Sequences of *Asaia* sp. shared 99% similarity with sequences of *Asaia* sp. (MN094403, MN094402, and MK598732) from China.

**Table 3:** Incidences and prevalences of gut microbes in wild-caught sand flies

Sand fly spp.	*Halomonas*	*Enterobacter*	*Klebsiella*	*Olivibacter*	*Pantoea*	*Botryotrichum*	*Acetobacter*	*Stenotropho-monas*	*Rhizobiales*	*Asaia*
*Ph. aculeatus*	0	0	0	0	0	0	0	0	0	0
*Ph. duboscqi*	0	0	0	0	0	0	0	0	0	0
*Ph. guggisbergi*	0	0	0	0	0	0	0	0	1 (0.9%)	1 (0.9%)
*Ph. martini*	0	0	0	0	0	0	0	0	0	0
*Ph. mireillae*	0	0	0	0	0	0	0	0	0	0
*Ph. orientalis*	0	0	0	0	0	0	0	0	0	0
*Ph. saevus*	0	0	0	0	0	0	0	0	0	0
*S. adleri*	0	0	0	0	0	0	1 (0.8%)	0	0	0
*S. africana*	0	0	0	0	1 (2.3%)	0	0	0	0	0
*S. antennata*	0	0	0	0	0	0	0	0	0	0
*S. bedfordi*	0	0	0	0	0	0	0	0	0	0
*S. clydei*	0	1 (0.2%)	1 (0.2%)	1 (0.2%)	1 (0.2%)	0	2 (0.5%)	2 (0.5%)	0	0
*S. ingrami*	0	0	0	0	0	0	0	0	0	0
*S. schwetzi*	2 (0.8%)	0	2 (0.8%)	0	0	0	0	0	0	0
*S. squamipleuris*	1 (0.3%)	0	1 (0.3%)	0	0	1 (0.3%)	0	0	0	0
*S. dreyfussi*	0	0	1 (100.0%)	0	0	0	0	0	0	0
Total	3 (0.2%)	1 (0.1%)	5 (0.3%)	1 (0.1%)	2 (0.1%)	1 (0.1%)	3 (0.2%)	2 (0.1%)	1 (0.1%)	1 (0.1%)

The gut bacteria identified from the colony sand flies, also from *Arsenophonus* primers included *Klebsiella* sp. (GenBank accessions OR704201-OR704203) that shared 99% 16S rRNA sequence identity with *Klebsiella pneumoniae* (CP104678), *Ochrobactrum* sp. (GenBank accession OR704188) that shared 99% similarity with *Ochrobactrum intermedium* from Japan (MT649859) and South Arabia (KY194745), *Pantoea* sp. (GenBank accessions OR704190–OR704191) that shared 99% similarity to sequences of *Pantoea septica* (GenBank: KF913782, KF913784 to KF913786), *Raoultella* sp. (GenBank accession OR704192) that shared 99% similarity with *Raoultella ornithinolytica* (GenBank: MT071372) from China, and *Tatumella* sp. (GenBank accession; OR704196–OR704199) that shared 99% with *Tatumella ptyseos* (GenBank: MN367128) from the USA. Among the colony *Ph. duboscqi* specimens, we detected *Serratia* sp. symbiont sequences using the *Arsenophonus* primers (GenBank accessions OR704204–OR704210) with 99% identity to a *Serratia marcescens* from Ethiopia (GenBank: MN006026).

### Prevalence of *Rickettsia africae* and key endosymbionts in sand flies

We detected DNA of the pathogen *R. africae* in 0.7% (11/1700; 95% CI 0.3–1.2%),of wild-caught sand flies, predominantly in *Ph. martini* (4.7%, 5/106) and *Ph. guggisbergi* (1.8%, 2/112), as well as in *S. schwetzi*, *S. clydei*, and *Sergentomyia* sp. (Table 4). *Wolbachia* spp. were the most prevalent endosymbionts (8.5%, 145/1700; 95% CI 7.2–9.9%), followed by *Spiroplasma* sp. (1.4%, 23/1700; 95% CI 0.9–2.0%), *Rickettsia* endosymbionts (0.7%, 12/1700; 95% CI 0.4–1.2%), *Cardinium* sp. (0.4%, 6/1700; 95% CI 0.1–0.8%), and *Tubulinosema* sp. (0.1%, 1/1700; 95% CI 0.0–0.3%). Notably, among wild-caught males we detected *R. africae* DNA in one *Ph. martini* male and *Rickettsia* endosymbiont DNA in two males (*S. clydei* and *S. schwetzi*), whereas no males were positive for *Wolbachia*, *Spiroplasma*, *Cardinium*, or *Tubulinosema*.

**Table 4:** Incidences and prevalences of gut *R. africae* and endosymbionts in wild-caught sand flies

	Pathogen	Endosymbiont genera				
Sand fly spp.	*R. africae*	*Wolbachia*	*Rickettsia*	*Spiroplasma*	*Cardinium*	*Tubulinosema*
*Ph. aculeatus*	0	0	0	0	0	0
*Ph. duboscqi*	0	0	0	0	0	0
*Ph. guggisbergi*	2 (1.8%)	82 (73.2%)	4 (3.6%)	0	0	0
*Ph. martini*	5 (4.7%)	2 (1.9%)	0	0	0	0
*Ph. mireillae*	0	12 (92.3%)	0	0	0	0
*Ph. orientalis*	0	0	0	0	0	0
*Ph. saevus*	0	18 (48.6%)	4 (10.8%)	0	0	0
*S. adleri*	0	1 (0.8%)	0	0	0	0
*S. africana*	0	3 (7.0%)	1 (2.3%)	0	0	0
*S. antennata*	0	5 (6.8%)	0	5 (6.8%)	0	0
*S. bedfordi*	0	6 (7.6%)	1 (1.3%)	0	0	0
*S. clydei*	2 (0.5%)	3 (0.7%)	1 (0.2%)	2 (0.5%)	2 (0.5%)	1 (0.2%)
*S. ingrami*	0	0	0	0	0	0
*S. schwetzi*	1 (0.4%)	6 (2.3%)	1 (0.4%)	14 (5.3%)	1 (0.4%)	0
*S. squamipleuris*	1 (0.3%)	6 (1.6%)	0	2 (0.5%)	2 (0.5%)	0
*S. dreyfussi*	0	1 (100.0%)	0	0	1 (100.0%)	0
Total	11 (0.6%)	145 (8.5%)	12 (0.7%)	23 (1.4%)	6 (0.4%)	1 (0.1%)

At the species level, *Wolbachia* prevalences were particularly high in some *Phlebotomus* species, including *Ph. saevus* (48.6%), *Ph. guggisbergi* (73.2%), and *Ph. mireillae* (92.3%) (Table 4), contrasting with the generally low prevalences of other heritable endosymbionts and suggesting that *Wolbachia* is established in specific sand fly lineages in our study area.

### Molecular identification of *Leishmania* parasites

**Fig. 5:**
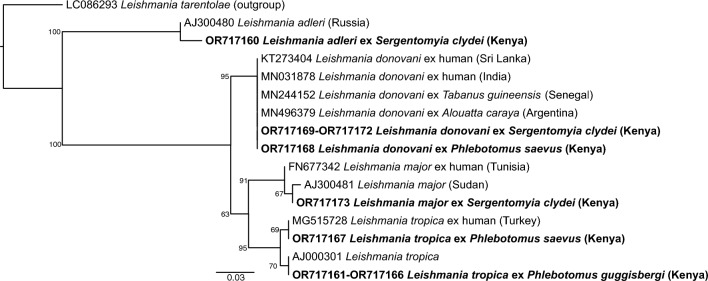
Maximum-likelihood tree of *Leishmania* ITS1 sequences. Sequences obtained in this study are in bold. Bootstrap support values (percentages) based on 1000 replicates are shown at major nodes. The branch length scale bar represents substitutions per site

We identified *Leishmania donovani* (GenBank accessions OR717168–OR717172), *Leishmania tropica* (GenBank accessions OR717161–OR717167), *Leishmania adleri* (GenBank accession OR717160), and *Leishmania major* (GenBank accession OR717173) from sand flies analyzed in this study (Fig. 5). The *L. donovani* (*n* = 5) sequences shared 100% similarity with *L. donovani* from Sri Lanka (GenBank: KT273404), Senegal (GenBank: MN244152), India (GenBank: MN031878), and Argentina (GenBank: MN496379). The *L. tropica* sequences from sand flies collected from Nguruman shared 100% identity with other *L. tropica* sequences (GenBank: MG515728, AJ000301), while *L. adleri* shared 99% identity with a *L. adleri* sequence from Russia (GenBank: AJ300480).

### Leishmania DNA in sand flies

We detected *Leishmania* DNA in 4.0% (50/1266) of the wild-caught female sand flies screened for *Leishmania* (Table 5). In sand flies from Nguruman, we detected *L. donovani* (5.6%, 47/844), *Leishmania adleri* (0.2%, 2/844) and* Leishmania tropica* (0.1%, 1/844). Generally, *L. donovani* was the most prevalent in three *Phlebotomus* sand fly species, *Ph. martini* (8.6%, 5/58), *Ph. orientalis* (13.3%, 2/15) and *Ph. saevus* (12.0%, 3/25), as well as in *Sergentomyia* species, *S. clydei* (6.1%, 17/278) and *Sergentomyia* sp. (3.9%, 13/338). In Gilgil, we found *L. tropica* (10.0%, 11/110) and *Leishmania major* (0.9%, 1/110), the latter only in *Ph. guggisbergi*.

**Table 5:** Detection rates of sand fly species in sampling areas and *Leishmania* DNA in sand fly species

	Parasites	*Ph. guggisbergi*	*Ph. martini*	*Ph. saevus*	*Ph. orientalis*	*S. adleri*	*S. africanus*	*S. bedfordi*	*S* *clydei*	*S. squamipleuris*
Sampling areas		*n* = 110	*n* = 58	*n* = 25	*n* = 15	*n* = 84	*n* = 32	*n* = 70	*n* = 278	*n* = 338
Nguruman	*L. donovani*	0	10.0% (5/50)	12.0% (3/25)	13.3% (2/15)	4.8% (4/84)	6.3% (2/32)	1.4% (1/70)	6.1% (17/278)	3.9% (13/338)
Nguruman	*L. tropica*	0	0	4.0% (1/25)	0	0	0	0	0	0
Nguruman	*L. adleri*	0	0	0	0	0	0	0	0.4% (1/278)	0.3% (1/338)
Gilgil	*L. tropica*	10.0% (11/110)	–	0	–	–	–	–	–	–
Gilgil	*L. major*	0.9% (1/110)	–	0	–	–	–	–	–	–
Parasites prevalence										
	*L. donovani*	0	8.6%	12.0%	13.3%	4.8%	6.3%	1.4%	6.1%	3.9%
	*L. tropica*	10.0%	0	4.0%	0	0	0	0	0	0
	*L. adleri*	0	0	0	0	0	0	0	0.4%	0.3%
	*L. major*	0.9%	0	0	0	0	0	0	0	0

### Associations between endosymbionts and *Leishmania* presence

We detected *Leishmania* DNA in significantly higher proportions of wild-caught sand flies with *Rickettsia* DNA (including both the *Rickettsia* endosymbionts and *Rickettsia africae*) (OR = 10.17; 95% CI [3.16, 28.39]; *P* = 0.0001). When considering only *R. africae*, this association was not significant (OR = 2.67; 95% CI [0.061, 19.28]; *P* = 0.34). However, there was still a positive association between *Rickettsia* endosymbionts and *Leishmania* (OR = 20.31, 95% CI [4.93, 77.03], *P* < 0.0001). The positive association between *Rickettsia* endosymbionts and *Leishmania* was also significant among *Phlebotomus* sand flies (OR = 13.54; 95% CI [2.33, 78.88]; *P* = 0.0017). Within individual *Phlebotomus* species, point estimates were elevated but confidence intervals were wide and included 1.0 due to small sample sizes: *Ph. saevus* (OR = 13.28; 95% CI [0.66, 295.46]; *P* = 0.05), *Ph. guggisbergi* (OR = 9.41; 95% CI [0.62, 143.19]; *p* = 0.056). No association was detected among *Sergentomyia* (OR = 11.93; 95% CI [0.22, 151.92]; *P* = 0.11). Similarly, we found a significant association between *Wolbachia* endosymbionts and *Leishmania* (OR = 2.46, 95% CI [1.17, 4.79]; *P* = 0.011) in wild-caught sand flies, but there was no association within either *Phlebotomus* (OR = 1.76; 95% CI [0.68, 4.59]; *P* = 0.27) or *Sergentomyia* (*P* = 1) genera.

## Discussion

Our results show that Kenyan sand flies harbor diverse microbiota, including heritable endosymbionts (*Rickettsia*, *Wolbachia*, *Spiroplasma*, *Cardinium*), gut-associated bacteria (*Serratia*, *Ochrobactrum*, *Asaia*), and a microsporidian (*Tubulinosema* sp.; Family: *Tubulinosematidae*). This diversity mirrors findings in other sand fly populations [48, 49] and suggests a complex microbial community potentially influencing vector competence. Furthermore, we found that sand flies with *Rickettsia* endosymbionts were more likely to also have *Leishmania* DNA, particularly in *Phlebotomus* sand flies of subgenus *Larroussius* (*Ph. guggisbergi*) and *Paraphlebotomus* (*Ph. saevus*). Rather than implying that *Rickettsia* directly enhances *Leishmania* growth and survival, we interpret this pattern as consistent with an association between *Rickettsia* infection and conditions that favor *Leishmania* establishment, akin to the positive association between *Sodalis* endosymbionts and trypanosomes in tsetse flies [50].

Alternatively, environmental/physiological factors that favor *Rickettsia* endosymbiont persistence in sand flies may also favor *Leishmania* survival. Such potential symbiont–parasite interactions warrant deeper investigation, and our cross-sectional data do not allow inference of causality. The significant associations between endosymbionts and *Leishmania* observed in our study align with emerging evidence that sand fly microbiota composition is associated with *Leishmania* susceptibility [51], and our finding of *Rickettsia* and *Wolbachia* co-occurring with *Leishmania* is consistent with recent reports of *Wolbachia* and *Rickettsia* coinfections in *Sergentomyia* species [52], suggesting complex tripartite interactions that merit investigation. Together, these findings situate our data within a growing body of evidence that sand-fly microbial communities influence parasite dynamics across genera and continents, while the underlying mechanisms remain to be elucidated.

Recent metagenomic work supports both positive and negative interactions between *Rickettsia* and *Leishmania*. For instance, Tom et al. (2025) found abundant *Rickettsia* in nonvector *Sergentomyia babu* but minimal levels in vector-competent *Ph. argentipes* [53], though whether this between-species difference reflects a functional relationship remains to be tested experimentally. Conversely, our positive associations in Kenyan sand flies may indicate strain- or species–specific facilitative effects, underscoring the complexity of *Rickettsia*–*Leishmania* relationships across sand-fly taxa. Furthermore, Itokawa et al. (2025) reported coinfection of *Sergentomyia squamirostris* with *Wolbachia* and Torix-group *Rickettsia* (“Candidatus Tisiphia”), providing the first complete genome of this clade from sand flies [52]. Such coinfections demonstrate that *Rickettsia* lineages are widespread and may interact with other symbionts within the same host.

This study provides the first record of *R. africae* in two vectors of leishmaniasis, *Ph. martini* and *Ph. guggisbergi,* and three biting-nuisance sand flies [54] of genus *Sergentomyia*, *S. clydei*, *S. schwetzi*, and *Sergentomyia* sp., expanding its known ecological range beyond ticks [55–57]. Notably, *S. clydei* has also been found to harbor *Leishmania* DNA and feed on humans in northern Kenya [4], suggesting potential involvement in *Leishmania* transmission cycles.

Detection of *R. africae* in both males and nonblood-fed females across multiple sand fly species is consistent with infection rather than recent blood-meal carry-over and raises the possibility of vertical transmission, similar to other *Rickettsia*–insect associations [58–60]. However, we cannot rule out repeated horizontal acquisition in sand flies. These occurrences of *R. africae* in sand flies could be attributed to the fact that wildlife are in free range conservancies in these areas of East Pokot, Kajiado West and Gilgil sub-counties, with likely active transmission of *R. africae*. Given the overlap of *R. africae* and *Leishmania* foci in our study areas, clinicians should consider both infections in differential diagnoses of febrile illness, as ATBF is frequently under-diagnosed in rural areas where clinical presentations overlap with other febrile illnesses [55, 61]. Targeted sand-fly control for leishmaniasis could therefore offer dual benefits by also reducing potential *R. africae* transmission.

In particular, detection of *R. africae* DNA in male *Ph. martini*, a nonblood-feeding sex, reinforces the interpretation that at least some infections are not due to recent vertebrate blood meals. However, the low prevalence of *R. africae* in males and the absence of *Wolbachia* and other symbionts in males do not fit the classical pattern of a common vertically transmitted endosymbiont, and the transmission route of *R. africae* in sand flies therefore remains unresolved. Taken together, the occurrence of *R. africae* across six sand fly species from three counties indicates that this pathogen is widespread in Kenyan sand flies and warrants further investigation of the role sand flies may play in the ecology and possible transmission of ATBF, including formal vector competence studies.

Rickettsiosis is neglected and cases could be going unreported in most African countries due to lack of knowledge, suspicion, and effective diagnosis in affected poor rural areas [55, 61]. Although our associations of *R. africae* to *Leishmania* were not significant, *R. africae* could have been acquired by sand flies zoonotically. *Anopheles* mosquitoes [59] and tsetse flies [60] have similarly also been shown to harbor pathogenic *Rickettsia*. More research is needed to determine the role of sand flies in ATBF transmission.

Although sand flies are known to host diverse microbial pathogens [48, 49], their broader microbiome remains poorly characterized [62], as does their potential role in modulating human pathogens, especially *Leishmania*, during its development in the sand fly. We highlight the presence of diverse endosymbionts in primary leishmaniasis vectors in Kenya, including *Rickettsia*, *Wolbachia*, *Spiroplasma*, *Cardinium*, and for the first time, a *microsporidian* species (*Tubulinosema* genus). Further studies are required to ascertain the nature of the symbiotic relationship of these endosymbionts with sand flies, which could potentially be targeted for development of biocontrol strategies against transmission of *Leishmania* parasites to humans and animals.

The known arthropod-associated *Wolbachia* endosymbionts [63] seem to preferably infect *Ph. guggisbergi*, *Ph. saevus*, and *Ph. mireillae*, which are found at high altitude*.* This pattern underscores the complex environmental, geographic, and ecological factors that shape endosymbiont distribution in sand-fly populations. Moreover, similar to our finding with *Rickettsia* endosymbionts, *Wolbachia* also showed a significant positive association to *Leishmania* in sand flies when considering all genera, but this association was not significant within genera. A large-scale survey found *Wolbachia* in more than 45% of *Phlebotomus* and *Sergentomyia* populations in Spain and Morocco, with both A and B supergroup strains [63]. The consistent occurrence of *Wolbachia* in *Leishmania infantum* vectors across two continents supports our conclusion that these symbionts are globally widespread in sand flies and could modulate vector competence in ways yet to be resolved.

Low-prevalence taxa such as *Spiroplasma*, *Cardinium*, and the microsporidian *Tubulinosema* were also detected. These microbes, known in other insects to influence reproduction or immunity [64–69], were rare in our samples and may represent either transient gut residents or coextracted taxa derived from ingested material or environmental contamination [69–73]. Their potential roles in sand fly physiology warrant confirmation through deeper metagenomic sequencing. Clarifying whether these microbes are stable symbionts or transient environmental associates will refine our understanding of sand-fly microbial ecology.

We did not detect the endosymbiont *Arsenophonus*, but other gut bacteria of genera *Serratia*, *Tatumella*, *Raoultella*, *Klebsiella, Ochrobactrum, Botryotrichum*, *Enterobacter*, and *Olivibacter* did amplify using the *Arsenophonus* primers. In colony *Ph. duboscqi* sand flies, we found *Serratia*, a colony-destroying bacterium, along with gut bacteria including *Tatumella*, *Raoultella*, as well as *Klebsiella* and *Ochrobactrum* that opportunistically infected immunocompromised persons [74, 75]. Diverse gut bacteria, including *Botryotrichum*, *Enterobacter*, and *Olivibacter*, which are mostly known to be opportunistic pathogens [76, 77], also occur in wild sand flies. Because we relied on partial 16S rRNA sequences, which often cannot reliably discriminate among closely related species, we report most gut-associated taxa at the genus level, even where BLAST hits suggested candidate species, to avoid overinterpretation of taxonomic assignments.

Notably, *Klebsiella pneumoniae* is a well-known opportunistic pathogen in humans, frequently causing nosocomial infections, urinary tract infections, and infections of the respiratory tract predominantly [78]. Also included are *Ochrobactrum*, which has been found to cause infections including endocarditis and septicemia in immunocompetent hosts [77] and *Tatumella ptyseos*, an opportunistic pathogen causing human sepsis [79], are opportunistic pathogens and their role in sand fly biology is not yet known. Although we could not confirm whether these gut bacteria are symbiotic to sand flies, detection of *Asaia*, a genus of symbiont found to be associated with the malaria vector *An. stephensi* [80]*,* and bacteria that reduce *Leishmania* establishment through colonization resistance, such as *Ochrobactrum intermedium* [81], in a sand fly vector of CL, *Ph. guggisbergi*, could provide a promising strategy against CL transmission. Recent work positions *Ochrobactrum sp.* as a promising paratransgenic symbiont due to its transstadial persistence and transformability [82, 83], and findings by Marialva et al. (2025) suggest that microbiota differences between susceptible and nonsusceptible sand fly populations could be exploited to enhance natural refractoriness to *Leishmania* [51]. Together, these insights reinforce the potential of microbiome-based vector-control strategies.

Our study has several limitations that should be considered when interpreting the endosymbiont and pathogen prevalences. First, although sand flies were externally washed prior to dissection, DNA was extracted from intact thorax and abdomen without separating the gut, meaning that some low-prevalence bacteria may represent transient gut residents rather than stable, heritable symbionts. In addition, head and genitalia were processed separately for morphological identification rather than molecular screening, so symbionts concentrated in reproductive tissues may have been under-detected [84]. Second, although detection of *R. africae* in males and nonblood-fed females is consistent with true infection, the low prevalence in males and the absence of *Wolbachia* and other symbionts in males do not conform to classical patterns expected for common vertically transmitted endosymbionts. This may reflect low statistical power due to the smaller male sample size (434 males versus 1266 females), sex-biased symbiont localization, or genuinely different transmission dynamics for *R. africae* compared to typical heritable symbionts. Finally, sample sizes for some sand fly species were modest, leading to wide confidence intervals for certain prevalence estimates. These findings therefore warrant confirmation in larger, tissue-targeted surveys that include reproductive tissues.

In our study areas of Gilgil and Nguruman, re-emerging foci of leishmaniasis, there is active transmission of *Leishmania*. We detected *L. donovani* DNA in *Ph. martini*, *Ph. orientalis* and several *Sergentomyia* species in Nguruman and a high prevalence of *L. tropica* in *Ph. guggisbergi* in Gilgil sub-county. This could be attributed to the fact that *Ph. guggisbergi* dwell mainly in rock crevices and caves inhabited by rock hyraxes that are known reservoirs of *L. tropica* [85]. We also found *L. tropica* for the first time in the newly collected *Ph. saevus* in Nguruman. Although *Ph. orientalis*, a species associated with cracked vertisols in dry seasons [6, 86], was rare during the rainy-season sampling, its detection with *L. donovani* DNA indicates its potential importance in *L. donovani* transmission in Nguruman. Although we found no *Leishmania* DNA in sand flies from East Pokot, we found *Ph. martini*, probably the main vector of VL in the region, also detected with *R. africae* and *Rickettsia* endosymbiont DNA.

## Conclusions

In this study, we identified diverse microbes associated with sand flies, some of which are closely related to microbes that have been used to control vector-borne diseases in other arthropods (e.g., *Wolbachia*), providing a step toward identifying and developing novel strategies to reduce transmission of leishmaniasis, a disease whose spread is being reshaped by climate and environmental change [3] and by drug resistance and limited treatment options [8, 9]. Therefore, we recommend: (i) targeted investigations to test the vector competence of sand flies for ATBF transmission, given the detection of *R. africae* across multiple sand fly species and in male specimens; (ii) establishment of integrated vector surveillance programs that screen for both *Leishmania* parasites and *Rickettsia* pathogens in endemic areas, particularly in the Rift Valley region where both leishmaniasis and rickettsial diseases co-occur; (iii) physician awareness of potential sand fly-borne rickettsiosis in patients presenting with febrile illness in leishmaniasis-endemic areas; and (iv) further investigations into the potential modulatory effects of *Rickettsia* and *Wolbachia* endosymbionts on sand fly biology and *Leishmania* parasite development, which could inform novel biocontrol strategies against leishmaniasis transmission.

## Supplementary Information

Additional file 1.Table S1. GPS coordinates for sand fly trapping sites, altitudes and ecological descriptions. Table S2. List of pathogens, endosymbionts, and gut bacteria 16S gene sequences obtained from this study

Additional file 2.Figure S1. Laboratory screening workflow

## Data Availability

Data is provided within the manuscript or supplementary information files. All sequence data generated in this study have been deposited in the GenBank database under accession numbers: OR731090-OR731091 (**R. africae* ompB*), OR704165-OR704178 (**Rickettsia** spp. 16S), OR704180-OR704181 (**Acetobacter** sp. 16S), OR704182-OR704183 (**Asaia** sp. 16S), OR704184-OR704185 (**Enterobacter** sp. 16S), OR704186-OR704187 (**Halomonas** sp. 16S), OR704188 (**Ochrobactrum** sp. 16S), OR704189 (**Olivibacter** sp. 16S), OR704190-OR704191 (**Pantoea** sp. 16S), OR704192 (**Raoultella** sp. 16S), OR704193 (**Rhizobiale** sp. 16S), OR704194-OR704195 (**Stenotrophomonas** sp. 16S), OR704196-OR704199 (**Tatumella** sp. 16S), OR704200-OR704203 (**Klebsiella** sp. 16S), OR704204-OR704210 (**Serratia** sp. 16S), OR704211-OR704217 (**Spiroplasma** spp. 16S), OR704218-OR704292 (**Wolbachia** sp. 16S), OR704293 (**Cardinium** sp. 16S), OR717175 ( **Tubulinosema** sp. 16S), OR717160 (**Leishmania adleri** ITS1), OR717161-OR717167 (**Leishmania tropica** ITS1), OR717168-OR717172 (**Leishmania donovani** ITS1), OR717173 (**Leishmania major** ITS1), OR671474-OR671476 (**Ph. orientalis** COI), OR671477 (**Ph. aculeatus** COI), OR671478-OR671481 (**Ph. saevus** COI), OR671482-OR671484 (**Ph. mireillae** COI), OR671485-OR671487 (**S. antennata** COI), OR671488-OR671489 (**S. schwetzi** COI), OR671490-OR671493 (**Ph. martini** COI), OR671494-OR671497 (**S. clydei** COI), OR671498 (**S. dreyfussi** COI), OR671499-OR671503 (**Sergentomyia** sp. COI), OR726348 (**Ph. guggisbergi** COI), OR731096-OR731097 (**Ph. orientalis* cyt b*), OR731098-OR731099 (**Ph. saevus cyt* b*), OR731100-OR731103 (**Ph. mireillae* cyt b*). OR731104 (**S. africana* cyt b*), OR731105 (**S. antennata* cyt b*), OR731106-OR731116 (**S. schwetzi* cyt b*), OR731117-OR731122 (**Ph. martini* cyt b*), OR731123-OR731124 (**S. clydei* cyt b*), OR731125 (**S. dreyfussi* cyt b*), OR731126-OR731129 (**Sergentomyia** sp. *cyt b*), OR731130- OR731132 (**Ph. duboscqi* cyt b*), OR731133 (**Ph. guggisbergi* cyt b*). All other data supporting the findings of this study are available within the article and its supplementary information files. Additional data are available from the corresponding authors upon reasonable request.

## Abbreviations

ATBF: African tick-bite fever
CI: Confidence interval
CL: Cutaneous leishmaniasis
DNA: Deoxyribonucleic acid
GPS: Geographic positioning system
HRM: High-resolution melting
*L.*: *Leishmania*
MIC: Magnetic Induction Cycler
*ompB*: Outer membrane protein B
OR: Odds ratio
PCR: Polymerase chain reaction
*Ph.*: *Phlebotomus*
rRNA: Ribosomal ribonucleic acid
*R.*: *Rickettsia*
*S.*: *Sergentomyia*
VL: Visceral leishmaniasis
