# Supplementary material for: Sand fly endosymbionts in Kenya: Rickettsia and Wolbachia associations with Leishmania and detection of Rickettsia africae
Source: Parasit Vectors. 2026 Feb 11;19:117. doi: 10.1186/s13071-026-07283-7 (PMC12997755; doi:10.1186/s13071-026-07283-7)
Supplement: Supplementary file 1 — Additional file 1.Table S1. GPS coordinates for sand fly trapping sites, altitudes and ecological descriptions. Table S2. List of pathogens, endosymbionts, and gut bacteria 16S gene sequences obtained from this study [file 13071_2026_7283_MOESM1_ESM.docx]

**Supplementary data**

**Supplementary Table S1. GPS coordinates for sand fly trapping sites, altitudes and ecological descriptions**

| **Study areas** | **Location** | | | | **Ecotypes** |
| --- | --- | --- | --- | --- | --- |
| **County** | **Trapping site** | **South** | **North** | **Altitude (m)** |  |
| **Baringo** | **East Pokot sub-County** | |  |  |  |
|  | Chesakam | 0.01419 | 36.01557 | 875 | Termite mound & Acacia |
|  | Kamsuk | 0.94725 | 35.96565 | 885 | Termite mound & Acacia |
|  | Lorwatum | 0.02337 | 35.98594 | 870 | Termite mound & Acacia |
| **Nakuru** | **Gilgil sub-County** | |  |  |  |
|  | Njeru | -0.582909 | 36.083596 | 2060 | Rocky cliffs & Hyraxes |
|  | Jaica | -0.568579 | 36.237019 | 1913 | Rocky cliffs & Hyraxes |
|  | Thugunui | -0.386075 | 36.26566 | 2198 | Rocky cliffs & Hyraxes |
| **Kajiado** | **Kajiado West sub-County** | |  |  |  |
|  | Birika | -1.862122 | 36.175471 | 682 | Termite mound & Acacia |
|  | Empaleki | -1.860102 | 36.106721 | 652 | Termite mound & Acacia |
|  | Enchanipus | -1.848106 | 36.107696 | 662 | Termite mound & Acacia |
|  | Kirine | -1.830574 | 36.10271 | 655 | Termite mound & Acacia |
|  | Olomanyatta | -1.864282 | 36.118434 | 659 | Termite mound & Acacia |
|  | Olosinyai | -1.875549 | 36.157326 | 647 | Termite mound & Acacia |
|  | Nkonyoro | -1.831591 | 36.171058 | 693 | Rock crevices & Acacia |
|  | Shompole | -2.102900 | 36.116083 | 644 | Termite mound & Acacia |

**Supplementary Table S2. List of pathogens, endosymbionts, and gut bacteria 16S gene sequences obtained from this study.**

| **Microorganism** | **Accession** | **Host sand fly** | **Collection area** |
| --- | --- | --- | --- |
| *Rickettsia africae* | OR704165 | *Sergentomyia* sp. | East Pokot |
|  | OR704166 | *Ph. martini* | East Pokot |
|  | OR704167 | *S. schwetzi* | East Pokot |
|  | OR704168 | *S. clydei* | East Pokot |
|  | OR704169 | *Ph. guggisbergi* | Gilgil |
|  | OR704170 | *Ph. martini* | Kajiado West |
|  | OR704171 | *S. clydei* | Kajiado West |
|  | OR704172 | *Ph. guggisbergi* | Gilgil |
| *Rickettsia* sp. | OR704173 | *Ph. saevus* | Kajiado West |
|  | OR704174 | *Ph. saevus* | Kajiado West |
|  | OR704175 | *S. clydei* | East Pokot |
|  | OR704176 | *S. schwetzi* | East Pokot |
|  | OR704177 | *S. bedfordi* | Kajiado West |
|  | OR704178 | *S. africana* | Kajiado West |
| *Wolbachia* sp. | OR704218 | *Ph. saevus* | Kajiado West |
|  | OR704219-OR704231 | *Ph. guggisbergi* | Gilgil |
|  | OR704232-OR704233 | *Ph. mireillae* | Gilgil |
|  | OR704234-OR704263 | *Ph. guggisbergi* | Gilgil |
|  | OR704264 | *Ph, mireillae* | Gilgil |
|  | OR704265 | *Ph. saevus* | Gilgil |
|  | OR704266-OR704285 | *Ph. guggisbergi* | Gilgil |
|  | OR704286 | *Ph. saevus* | Gilgil |
|  | OR704287-OR704288 | *Ph. mireillae* | Gilgil |
|  | OR704289 | *S. africana* | East Pokot |
|  | OR704290 | *Ph. guggisbergi* | Gilgil |
|  | OR704291-OR704292 | *S. africana* | East Pokot |
| *Spiroplasma* sp. | OR704211-OR704212 | *S. schwetzi* | East Pokot |
|  | OR704213 | *Sergentomyia* sp*.* | East Pokot |
|  | OR704214-OR704217 | *S. schwetzi* | East Pokot |
| *Cardinium* sp. | OR704293 | *S. dreyfussi* | East Pokot |
| *Tubulinosema* sp. | OR717175 | *S. clydei* | East Pokot |
| *Halomonas* sp. | OR704186 | *S. schwetzi* | East Pokot |
|  | OR704187 | *Sergentomyia* sp. | East Pokot |
| *Acetobacter* sp. | OR704180-OR704181 | *S. clydei* | Kajiado West |
| *Asaia* sp. | OR704182-OR704183 | *Ph. guggisbergi* | Gilgil |
| *Enterobacter* sp. | OR704184 | *S. clydei* | East Pokot |
|  | OR704185 | *Ph. duboscqi* | Colony |
| *Ochrobactrum* sp. | OR704188 | *Ph. duboscqi* | Colony |
| *Olivibacter* sp. | OR704189 | *S. clydei* | East Pokot |
| *Pantoea* sp. | OR704190 | *S. clydei* | East Pokot |
|  | OR704191 | *S. africana* | East Pokot |
| *Raoultella* sp. | OR704192 | *Ph. duboscqi* | Colony |
| *Rhizobiale* sp. | OR704193 | *Ph. guggisbergi* | Gilgil |
| *Stenotrophomonas* sp. | OR704194-OR704195 | *S. clydei* | East Pokot |
| *Tatumella* sp. | OR704196-OR704199 | *Ph. duboscqi* | Colony |
| *Klebsiella* sp. | OR704200 | *Sergentomyia* sp. | East Pokot |
|  | OR704201-OR704202 | *S. schwetzi* | East Pokot |
|  | OR704203 | *S. clydei* | East Pokot |
| *Serrati*a sp. | OR704204-OR704210 | *Ph. duboscqi* | Colony |
