# Supplementary figures and images for: Sand fly endosymbionts in Kenya: Rickettsia and Wolbachia associations with Leishmania and detection of Rickettsia africae
Source: Parasit Vectors. 2026 Feb 11;19:117. doi: 10.1186/s13071-026-07283-7 (PMC12997755; doi:10.1186/s13071-026-07283-7)

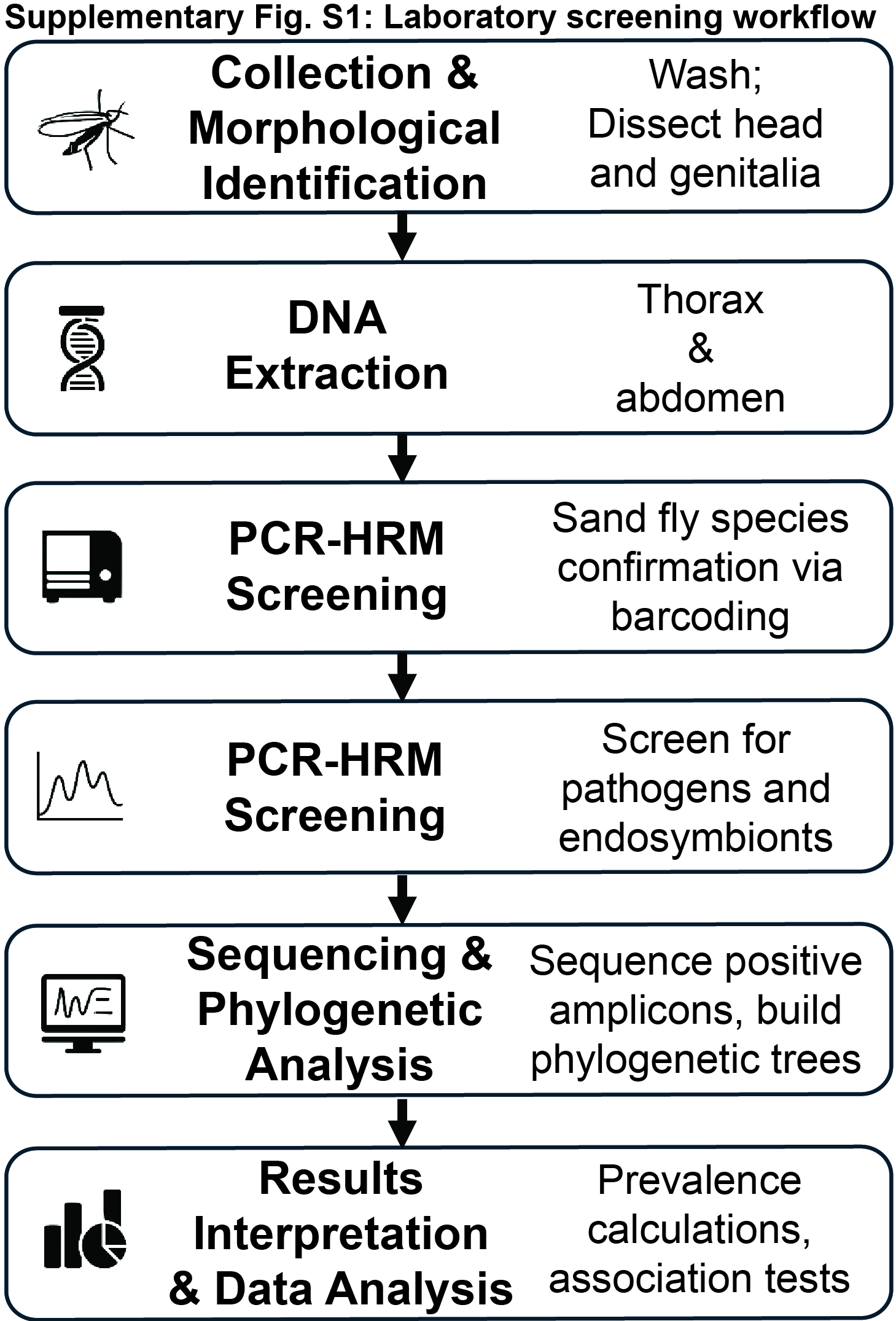

Supplement: Supplementary file 2 — Additional file 2.Figure S1. Laboratory screening workflow [file 13071_2026_7283_MOESM2_ESM.tif]
